# Editorial: Oropharyngeal and nasopharyngeal microflora: new horizons in human immunity and clinical practice

**DOI:** 10.3389/fimmu.2023.1206747

**Published:** 2023-06-01

**Authors:** Chenyi Zhu, Xi Chen, Zhiyuan Ye, Xiaomei Wang, Qiang Wang

**Affiliations:** Institute of Infection, Immunology and Tumor Microenvironment, Hubei Province Key Laboratory of Occupational Hazard Identification and Control, Medical College, Wuhan Asia General Hospital, Wuhan University of Science and Technology, Wuhan, China

**Keywords:** oropharyngeal and nasopharyngeal microflora, immunology, clinical practice, 16S rRNA, inflammatory factor

Research is increasingly showing the wide-ranging influence of the oropharyngeal/nasopharyngeal microbiota on acute and/or chronic respiratory infections. Once oral microorganisms have the opportunity to be transferred to the circulatory system or digestive system, the occurrence and development of respiratory infections and systemic diseases will be tightly associated with the oral microecosystem. The interactions between oropharyngeal/nasopharyngeal microbiota and host mucosal immunity could represent the primary defensive layer against potential pathogens. Alternatively, these dynamic communities could stimulate inflammatory factors, which further induce oral and gut dysbiosis that is linked to many other diseases.

Four research papers were published within the Research Topic “*Oropharyngeal and nasopharyngeal microflora: new horizons in human immunity and clinical practice*”. The relationship between the oral and nasopharyngeal microbiota and the occurrence and development of disease, as well as nasopharyngeal metagenomic research, were introduced and discussed, providing a reference for researchers and contributing to improved clinical practice.

In the study by Bertuccioli et al., the efficacy of Streptococcus salivarius K12 (S. salivarius K12) was investigated on healthy college students that were divided into a treatment group and a control group (placebo). The authors found that a relatively short-term S. salivarius K12 supplementation significantly increased the levels and secretion of sIgA in healthy subjects after a period of training, and suggested that S. salivarius K12 supplementation might be a promising candidate for increasing sIgA levels and optimizing mucosal microbial colonization and mucosal barrier function. The limitation of this study is that the sample size is insufficient, and further research on a larger population needs to be conducted.

Rajar et al. explored and compared the efficiency of different protocols combining host DNA depletion and microbial DNA extraction from nasopharyngeal aspirates of premature infants to profile the microbiome and drug resistance using whole metagenomic sequencing (WMS). Mol_ MasterPure, which is composed of host DNA depletion with MolYsis™ Basic5 and DNA extraction with MasterPure™ Gram Positive DNA Purification Kit, is a better extraction method for low microbial biomass samples with a high human DNA content. WMS could be used effectively to expand and improve the application for studying the respiratory system and other low-biomass microorganisms. However, we still need more comprehensive and extensive research to further optimize these approaches.

Dang et al. investigated the sputum flora characteristics in patients with chronic obstructive pulmonary disease (COPD) that exhibit frequent exacerbations in the clinically stable stage, so as to prevent and interfere with the progression of this phenotype. Sputum samples from patients with or without frequent exacerbations of COPD were collected during clinical stability and sequenced for 16S rRNA, which was then subjected to amplicon sequence variants (ASVs)-based microbiome analysis. The experiment proved for the first time that there was a positive correlation between the frequent exacerbation phenotype of COPD and the sputum microflora during the period of clinical stability, which provided a potential diagnosis and treatment target for subsequent research and clinical practice. The respiratory microbiota has provided a new perspective for exploring potential biomarkers for the recognition of COPD and its subtypes, but further research is needed on the detailed characteristics of the respiratory microbiota in patients with frequent and infrequent exacerbations of COPD in order to explore more iconic biomarkers.

Bartosik et al. sought to understand the effect of microbiota on the underlying inflammatory process and disease burden by measuring clinical parameters and collecting nasal samples (nasal mucosal fluid and microbiota swabs) in patients with chronic rhinosinusitis (CRS) without nasal polyps (CRSsNP) and with nasal polyps (CRSwNP), or N-ERD (NSAID-exacerbated respiratory disease) and in patients without CRS (disease controls). 16S rRNA gene amplicon sequencing was used to analyze the nasal microbiota, and the MSD (Meso Scale Discovery) platform was used to determine the levels of inflammatory cytokines in the nasal mucosal fluid. Their study suggested a strong association between increased Staphylococcus and decreased Corynebacterium colonization, as well as increased Type 2 inflammation and disease burden. This suggests that targeted improvement of the nasal microbiota can be used to reduce the burden in patients with non-steroidal anti-inflammatory drugs (N-ERD) and CRSwNP. It adds to our understanding of the roles of the nasal microbiota in controlling health and diseased states of the nasal mucosa.

Taken together, the research and interventions targeting the oropharyngeal/nasopharyngeal microbiota represent an emerging field of human immunology research and clinical practice. These articles are presented in different disease contexts and represent the importance of the oropharyngeal/nasopharyngeal microbiota from various perspectives. In addition, the current research progress and development direction were introduced in detail, which provided new ideas for the clinical application of oropharyngeal/nasopharyngeal microbiota. With the continuous development of microbial sequencing technology, the composition and functional characteristics of the oropharyngeal/nasopharyngeal microbiota can be analyzed in greater detail. Defining the diversity and function of the oropharyngeal/nasopharyngeal microflora might be beneficial for the timely prediction and diagnosis of diseases related to respiratory microbiota. Dynamic changes in the oropharyngeal/nasopharyngeal microbiota can make positive or negative contributions to the host’s immune system. A new era of oropharyngeal/nasopharyngeal microflora study will benefit patients in clinical practice.

## Author contributions

QW organized the writing of this review. CZ, XC, and ZY divided the remaining tasks of writing. XW revised the weak grammatical part of the article. All authors have read and agreed to the published version of the manuscript.

